# Supplementary material for: Controversies in the Surgical Treatment of Chronic Subdural Hematoma: A Systematic Scoping Review
Source: Diagnostics (Basel). 2022 Aug 25;12(9):2060. doi: 10.3390/diagnostics12092060 (PMC9498240; doi:10.3390/diagnostics12092060)
Supplement: Supplementary file 1 [file diagnostics-12-02060-s001.zip › diagnostics-1842559-supplementary.pdf]

Supplementary Table S1. "Levels of evidence and grades of recommendation, modified from Weigel et al. [5]."

LEVEL OF EVIDENCE	EXPLANATION	
LEVEL I	Evidence provided by one or more well designed RCTs.	
LEVEL II	Evidence provided by one or more well designed clinical studies such as prospective studies, case-control studies, etc..	
LEVEL III	Evidence provided by non-randomized retrospective studies, case series, case reports and expert opinion.	
**GRADE OF RECOMMENDATION**	**STRENGTH**	**TRANSLATION IN PRACTICE**
GRADE I	Strong recommendation based on class I evidence or consistent level II evidence.	Clinicians should follow the recommendation unless a clear and compelling rationale for an attentive approach exists.
GRADE II	Moderate recommendation based on level II evidence.	Clinicians should follow the recommendation but should remain sensitive to new information and patient's preference.
GRADE III	Weak recommendation based on level II evidence.	Clinicians should remain flexible but take into consideration experts opinion. Precision on a case-by-case basis.
